# Direct-to-implant breast reconstruction: retrospective analysis of 91 consecutive cases

**DOI:** 10.1007/s13304-025-02425-5

**Published:** 2025-10-14

**Authors:** Giuseppe Masciopinto, Tito Brambullo, Alberto Marchet, Andrea Varotto, Massimo Marini, Gian Paolo Azzena, Gloria Fanton, Vincenzo Vindigni

**Affiliations:** 1 Plastic Surgery Unit, Department of Neurosciences, University of Padua, via Giustiniani 2, Padova, Veneto Italy; 2 Breast Surgery Unit, Veneto Institute of Oncology, Padova, Italy; 3 Week Surgery Unit, Department of Surgical, Oncological and Gastroenterological Sciences, University Hospital of Padova, Padua, Italy; 4 Department of Psychiatry, Department of Neuroscience, Padua University Hospital, Padua, Italy; 5 Plastic Surgery Unit, Department of Surgery, Dell’Angelo Hospital, Venice, Italy

**Keywords:** Acellular dermal matrix, Breast cancer, Direct-to-implant, Hybrid breast reconstruction, Implant, Mastectomy

## Abstract

Implant-based reconstruction is the main post-mastectomy reconstructive method. With the introduction of acellular dermal matrices (ADM), direct-to-implant reconstruction has, in selected patients, replaced dual-stage technique with tissue expander. The objective of this study is to compare postoperative outcomes and quality of life of three DTI breast reconstruction techniques: pre-pectoral with ADM versus submuscular without ADM versus submuscular with ADM (dual plane). A retrospective study was conducted between January 2020 and April 2023. The enrolled patients were divided into three groups: patients who underwent mastectomy and pre-pectoral reconstruction with ADM (group A); patients treated with submuscular reconstruction without ADM (group B); and patients who underwent submuscular reconstruction with ADM (group C). Minor and major complications, revision rates, and capsular contracture rates were recorded, and patients underwent the BRECON-31 postoperative questionnaire. A total of 27 pre-pectoral reconstructions with ADM, 41 submuscular reconstructions without ADM, and 23 dual-plane reconstructions with ADM were performed. The incidence of major complications (*p* = 0.076) and severe capsular contracture (*p* = 0.048) was higher in group C. Group C achieved lower results than other groups for all BRECON-31 categories except for Recovery category. According to the study findings, the direct-to-implant breast reconstruction showed to be effective; however, the pre-pectoral technique with ADM does not seem to have superiority over the conventional muscle-pocket technique in terms of reducing complication rate and overall patient satisfaction.

Level of evidence: 4.

## Introduction

Implant-based breast reconstruction is the primary method of post-mastectomy reconstruction, covering 72.6% of reconstructions [1, 2]. This method is often preferred over autologous reconstruction due to shorter operative time, shorter hospital stay, faster recovery, absence of donor site comorbidities, and lower complication rates [3, 4].

Prosthetic reconstruction can be performed immediately at the time of mastectomy or in two stages [5, 6].

Especially in cases of nipple-sparing mastectomy (NSM), direct-to-implant (DTI) reconstruction techniques have proven useful in producing acceptable results with fewer interventions [7]. Initially, implant-based breast reconstruction was routinely performed in the subcutaneous plane in patients undergoing radical mastectomy with sacrifice of the pectoralis major muscle [8–10]. Following the introduction of conservative mastectomies, the implant is positioned under the pectoralis major muscle and/or the serratus muscle, with or without acellular dermal matrix (ADM) [11]. Support and coverage from the pectoralis major muscle lowered capsular contracture risk and reduced implant complications (e.g., implant visibility, palpability, and exposure and capsular contracture) [8]. However, muscle elevation led to issues such as animation deformities from muscle contraction, chest tightness, pain, spasms, and unnatural appearance of the breast [12]. For those reasons in recent years, the paradigm has changed further with the reintroduction of pre-pectoral reconstructions [13–16].

In this study, we aimed to compare three different surgical strategies for immediate prosthetic breast reconstruction to verify whether there were any differences in terms of complications and patient satisfaction.

The study population was divided based on the surgical pocket and on the use or not of ADM.

## Materials and methods

### Study design

A retrospective comparative study was conducted on 91 patients (105 breasts) who received mastectomy and direct-to-implant breast reconstruction (DTI) at our Clinic between January 2021 and April 2023.

All patients undergoing immediate implant-based reconstruction after unilateral and/or bilateral mastectomy for invasive breast tumors, ductal carcinoma in situ, and risk-reducing mastectomies were included.

Oncologic breasts presented tumors varying from extensive in situ cancer to invasive from T1 to T3 without direct invasion of skin or pectoral muscles, with cases of concurrent different histotypes and sizes in the same specimen. T4 tumors were excluded from direct implant reconstruction.

Patients undergoing reconstruction with tissue expanders, autologous reconstruction, hybrid reconstruction with implants and local flaps, and patients not undergoing reconstruction were excluded. Furthermore, patients with unstable medical or psychiatric conditions were excluded.

The study population was divided into three groups: Group A (*n* = 27) consisted of patients undergoing pre-pectoral reconstruction using acellular dermal matrix (ADM) Braxon^Ⓡ^ (Decomed, Venice, Italy) as complete coating, Group B (*n* = 41) comprised cases undergoing retro-pectoral reconstruction without ADM, and Group C (*n* = 23) included those undergoing retro-pectoral reconstruction with ADM-wrapped implants (dual-plane reconstruction). Unlike conventional dual-plane reconstructions where an ADM patch is placed between the lower edge of the pectoralis muscle (previously dissected at the level of its sterno-costal insertions) and the chest wall, a preformed ADM sheet completely envelops the implant in this study [17].

Patients were assigned to one of three groups based on the local conditions of the breast soft tissues at the end of the mastectomy. The thickness and perfusion of the skin flaps, their length, and the size of the implant were carefully evaluated, and then patients in clear good condition were randomly allocated to groups 1 or 2, reserving group 3 for the patients who presented adequate but suboptimal characteristics, particularly in cases such as skin-sparing mastectomy in large breasts, which may not perfectly align with the recommendations for direct implant reconstruction. This selection, far from being a rule, was based on the surgeon’s perspective and personal preferences.

Patient demographic and characteristic data were recorded. Surgical variables examined included procedure duration, laterality, type of mastectomy (nipple-sparing, skin-sparing, skin-reducing), implant size, nodal surgery (sentinel lymph node biopsy and/or axillary dissection), contralateral symmetrization, and assessment of mastectomy flap perfusion using indocyanine green angiography.

Patients were followed up with standard follow-up at 1–3–6 and 12 months, and standardized images were taken at each follow-up.

Postoperative complications were analyzed for each patient and categorized as minor (managed on an outpatient basis with dressings or ambulatory surgical procedures under local anesthesia) and major (requiring surgical intervention in the operating room).

Each patient was evaluated for capsular contracture during postoperative clinical follow-ups, classified according to Baker’s classification [18]. The incidence of early (within 2 months post-procedure) and late (after the second postoperative month) surgical revisions was also analyzed.

The Brecon-31 questionnaire was administered at least 6 months after the procedure to assess the patients’ perspective on the esthetic and functional outcomes of reconstruction [19].

All procedures performed in studies involving human participants were in accordance with the ethical standards of the institutional and/or national research committee and with the 1964 Declaration of Helsinki and its later amendments or comparable ethical standards. This study received approval from our local Ethics Committee (CET_ACEV code: 530n/AO/24).

### Operative technique

Group A involves the use of ADM for pre-pectoral reconstruction following mastectomy. In this procedure, the pre-shaped porcine dermis matrix BraxonⓇ is placed around the implant and held in place with Vicryl 3/0 sutures. The ADM implant unit is then positioned within the pocket and optionally stabilized to the muscle plane using Vicryl 2/0 anchoring stitches. Prior to implant insertion, suction drainage is always placed at the caudal portion of the pocket.

Group B involves submuscular reconstruction without the use of ADM. To prepare the implant pocket, the pectoralis major muscle is incised at its inferolateral free edge, and the submuscular plane is accessed. The sterno-costal insertions of the muscle are dissected from the 4 o'clock to the 8 o'clock positions of the chest wall, while keeping the muscle fascia intact. The entire belly or external fascia of the serratus anterior muscle was dissected to completely or nearly completely cover the implant under the muscle plane. In most cases, the surgeon preferred the first option.

The pectoralis major and serratus anterior muscles are sutured, as much as possible, with Vicryl 2/0. Suction drainage is always placed at the submuscular plane and subcutaneous plane.

Group C involves submuscular reconstruction with ADM (dual plane). The selected implant, as per the pre-pectoral technique, is completely enveloped by ADM BraxonⓇ and subsequently placed beneath the pectoralis major muscle after dissecting its sterno-costal insertions. The muscle can then be optionally secured to the anterior portion of the ADM with Vicryl 2/0. In this instance, no dissection of the serratus anterior muscle is carried out. Similar to patients in Group B, two suction drains are placed, one at the submuscular plane and one at the subcutaneous plane.

All the breast implants used in the surgical procedures were silicone-based, textured and teardrop shaped (MENTOR™ Contour Profile Gel™ Breast Implants, Mentor Worldwide LLC, Santa Barbara, California).

### Statistical analysis

Data were recorded using Microsoft Excel for Mac OS, Ver.16.45 (2021 Microsoft^Ⓒ^, Redmond, WA), and statistical analysis was performed using SPSS^Ⓡ^ Statistics for Mac OS, Ver.25 (IBM Corporation, Armonk, NY). Categorical variables were described using number and percentage, and continuous variables were described using mean, standard deviation, minimum, and maximum.

To analyze differences between groups, Fisher’s exact test was used for dichotomous variables, the Chi-square test was used for unordered categorical variables, and ANOVA was used for continuous variables.

Statistical significance was considered at *p* < 0.05, while values of *p* < 0.1 were considered statistically borderline.

## Results

The final study sample consists of 91 patients and 105 breasts. 14 patients underwent bilateral mastectomy with simultaneous prosthetic reconstruction (15.38%).

The socio-demographic and preoperative characteristics of the population are summarized in Table 1. These variables are homogeneous across the three study groups, with no statistically significant differences observed.

**Table 1:** Distributions of anthropometric variables and preoperative and perioperative risk factors across the three study groups

	Total	Group A	Group B	Group C	*p* value
Mean age (SD)	54.95 (± 10.08)	55.26 (± 9.574)	56.34 (± 10.88)	52.09 (± 8.964)	0.267
Mean BMI (SD)	22.98 (± 3.5)	22 (± 2.66)	23.13 (± 3.76)	24.17 (± 3.7)	0.181
Comorbidities	41/91 (45.1%)	11/27 (40.7%)	18/41 (42.9%)	12/23 (52.2%)	0.713
Smoking history	12/91 (13.2%)	2/27 (7.4%)	7/41 (18.1%)	3/23 (13%)	0.486
Prior surgery	16/91 (17.6%)	5/27 (18.5%)	5/41 (12.2%)	6/23 (26.1%)	0.361
Prior QUART	12/91 (13.2%)	4/27 (14.8%)	4/41 (9.8%)	4/23 (17.4%)	0.728
Neoadjuvant RT	3/91 (3.3%)	0/27 (0%)	1/41 (2.4%)	2/23 (8.7%)	0.268
Adjuvant RT	7/89 (7.9%)	3/26 (11.5%)	1/40 (2.5%)	3/23 (13%)	0.274
Neoadjuvant CT	11/91 (12.1%)	2/27 (7.4%)	6/41 (14.6%)	3/23 (13%)	0.661

In particular, the retrospective analysis did not reveal any significant difference in the incidence of comorbidity, defined as the presence of at least one systemic disease among arterial hypertension, diabetes, coagulation alterations, diseases of the hematopoietic system, and cardiac and circulatory diseases.

The surgical time increased with the complexity of surgery: Group A had an average duration of 155.37 ± 37.36 min, Group B of 182.46 ± 53.325 min, and Group C of 201.91 ± 43.11 min (*p = 0.003,* *F* = 6.367).

Overall, 66 patients (72.52%) underwent nipple-sparing mastectomy (NSM), 25 (27.47%) skin-sparing mastectomy (SSM), and 14 (15.38%) patients skin-reducing mastectomy (SRM). Analysis of the distribution of mastectomy types across the three study groups showed a higher rate of NSM in Groups A and B (80.6% and 61.7%, respectively) and a lower frequency in Group C (44.4%) with *p = 0.018.* SSM was more frequently performed in Group C (48.1%) and less in Groups A and B (9.7% and 19.1%, respectively) with *p* = 0.001. The distribution of SRM cases was more homogeneous across the three groups (9.7% in Group A, 19.1% in Group B, and 7.4% in Group C; *p* = 0.307).

The breast implants placed in Group A had a smaller volume compared to the other groups (*p* = 0.018, *F* 4.196) with a mean of 320.56 ± 95.26 cc compared to 395.13 ± 118.913 cc in Group B and 396.74 ± 120.544 cc in Group C.

The distributions of contralateral symmetrization mammaplasty (*p* = 0.473) and the use of indocyanine green angiography to check skin flap perfusion (*p* = 0.350) were homogeneous across the three groups.

Although not statistically significant (*p* = 0.341), the distribution of axillary lymph node dissections showed a higher rate in Group C (33.1%) compared to Groups A and B (22.2% and 24.4%, respectively). In addition, 8/10 (80%) of the cases in Group C with major complications and severe capsular contracture underwent axillary lymph node dissection, compared to 25% in Group A and 50% in Group B (Tables 2 and 3).

**Table 2:** Intraoperative variables

	Total	Group A	Group B	Group C	*p* value
Mean surgical time (SD)	179.34 (± 49.281)	155.37 (± 37.36)	182.46 (± 53.325)	201.91 (± 34.11)	0.003
Mean implant volume (SD)	373.17 (± 116.789)	320.56 (± 95.26)	395.13 (± 118.913)	396.74 (± 120.544)	0.018
Bilateral	14/91 (15.4%)	4/27 (14.8%)	6/41 (14.6%)	4/23 (17.4%)	1.00
NSM	66/105 (62.9%)	25/31 (80.6%)	29/47 (61.7%)	12/27 (44.4%)	0.018
SSM	25/105 (23.8%)	3/31 (9.7%)	9/47 (19.1%)	13/27 (48.1%)	0.001
SRM	14/105 (13.3%)	3/31 (9.7%)	9/47 (19.1%)	2/27 (7.4%)	0.307
Axillary dissection	25/91 (27.5%)	6/27 (22.2%)	10/41 (24.4%)	9/23 (33.1%)	0.341
Controlateral surgery	56/91 (61.5%)	14/27 (51.9%)	27/41 (65.9%)	15/23 (65.2%)	0.473
IG angiography	13/91 (14.3%)	6/27 (22.2%)	4/41 (9.8%)	3/23 (13%)	0.350

**Table 3:** Postoperative complications

Complications	Total	Group A	Group B	Group C	*p* value
Major	14/91 (15.4%)	3/27 (11.1%)	4/41 (9.8%)	7/23 (30.4%)	0.076
Minor	16/91 (17.6%)	2/27 (7.4%)	11/41 (26.8%)	3/23 (13%)	0.086
Infection	5/91 (5.5%)	0/27 (0%)	4/41 (9.8%)	1/23 (4.3%)	0.208
Dehiscence	12/91 (13.2%)	1/27 (3.7%)	7/41 (17.1%)	4/23 (17.4%)	0.232
Necrosis	14/91 (15.4%)	2/27 (7.4%)	6/41 (14.6%)	6/23 (26.1%)	0.208
Hematoma	3/91 (3.3%)	0/27 (0%)	1/41 (2.4%)	2/23 (8.7%)	0.268
Early seroma	10/91 (11%)	2/27 (7.4%)	3/41 (7.3%)	5/23 (21.7%)	0.175
Late seroma	2/91 (2.2%)	1/27 (3.7%)	0/41 (0%)	1/23 (4.3%)	0.499
Implant exposure	7/91 (7.7%)	1/27 (3.7%)	2/41 (4.9%)	4/23 (17.4%)	0.122
Implant loss	8/91 (8.8%)	1/27 (3.7%)	2/41 (4.9%)	5/23 (21.7%)	0.048

Statistical analysis of postoperative outcomes showed a higher rate of minor complications in Group B (11 cases, 26.8%) compared to Groups A (2 cases, 7.4%) and C (3 cases, 13%), with a Pearson Chi-square approaching significance (*p* = 0.086).

Major complications were more frequent in Group C (7/23 cases, 30.4%) compared to Group A (3/27 cases, 11.1%) and B (4/41 cases, 9.8%) with *p* = 0.076.

The incidence of prosthesis loss in the three groups, and subsequent replacement with expander or smaller volume prosthesis, was 1/27 (3.7%) in Group A, 2/41 (4.9%) in Group B, and 5/23 cases (21.7%) in Group C, with a *p* value of 0.048.

None of the individual complications observed in the study population showed a statistically significant distribution across the three groups.

The rates of severe capsular contracture (Baker grades III–IV) showed a statistically significant distribution across the three groups (*p* = 0.048) (Table 4). In Group A, there was 1 case of severe capsular contracture not subjected to surgical revision out of 27 (3.7%), while there were no cases of severe capsular contracture undergoing surgery. In Group B, there was 1 case of non-surgical severe contracture out of 41 (2.4%) and 1 case (2.4%) of severe capsular contracture undergoing surgery, totaling 2/41 cases (4.9%). Group C had the highest rate of severe capsular contracture: 2/23 cases (8.7%) not undergoing surgery and 3/23 cases (13%) undergoing surgery, totaling 5/23 cases (21.7%).

**Table 4:** Capsular contracture rate (Baker classification)

Capsular contracture	Total	Group A	Group B	Group C	*p* value
Grades I–II	83/91 (91.2%)	26/27 (96.3%)	39/41 (95.1%)	18/23 (78.3%)	0.048
Grades III–IV (conservative)	4/91 (4.4%)	1/27 (3.7%)	1/41 (2.4%)	2/23 (8.7%)	0.686
Grades III–IV (surgical revision)	4/91 (4.4%)	0/27 (0%)	1/41 (2.4%)	3/23 (13%)	0.055
Grades III–IV (total)	8/91 (8.8%)	1/27 (3.7%)	2/41 (4.9%)	5/23 (21.7%)	0.048

The rates of early surgical revision (within 60 days post-surgery) were 3/27 (22.2%) for Group A, 4/41 cases (9.8%) for Group B, and 7/23 cases (30.4%) for Group C, with a distribution nearing significance (*p* = 0.076).

The rates of late surgical revision (after 60 days), for severe capsular contracture or prosthetic rupture or displacement, did not show a statistically significant trend (*p* = 0.256): 1/27 (3.7%) in Group A, 3/41 cases (7.3%) for Group B and 4/23 (17.4%) in Group C (Table 5).

**Table 5:** Surgical revision rate

Surgical revision	Total	Group A	Group B	Group C	*p* value
Early	14/91 (15.4%)	3/27 (11.1%)	4/41 (9.8%)	7/23 (25.3%)	0.076
Late	8/91 (8.8%)	1/27 (3.7%)	3/41 (7.3%)	4/23 (17.4%)	0.256

The Brecon-31 questionnaire was completed by 63/91 (69.23%) patients, including 21/27 (77.77%) from Group A, 27/41 (65.85%) from Group B, and 15/23 (65.21%) from Group C (Table 6).

**Table 6:** Response rates to the BRECON-31 questionnaire categories

Category	Total	Group A	Group B	Group C
Self-image	61/63 (96.82%)	21/21 (100%)	27/27 (100%)	13/15 (86.66%)
Arm concerns	62/63 (98.41%)	21/21 (100%)	26/27 (96.29%)	15/15 (100%)
Intimacy	54/63 (85.71%)	19/21 (90.47%)	24/27 (88.88%)	11/15 (73.33%)
Satisfaction	53/62 (84.12%)	18/21 (85.71%)	23/27 (85.18%)	12/15 (80%)
Recovery	61/63 (96.82%)	20/21 (95.23%)	26/27 (96.29%)	15/15 (100%)
Self-consciousness	61/63 (96.82%)	21/21 (100%)	25/27 (95.59%)	15/15 (100%)
Expectations	55/63 (87.3%)	18/21 (85.71%)	23/27 (85.18%)	14/15 (93.33%)
Breast appearance	61/63 (96.82%)	21/21 (100%)	25/27 (95.59%)	15/15 (100%)
Summary score	40/63 (63.49%)	15/21 (71.42%)	17/27 (62.96%)	8/15 (53.33%)

Table 7 shows the mean scores of each Brecon-31 category across the three study groups. Although statistical analysis did not find significant differences, results indicate that pre-pectoral approach and submuscular approach with no ADM tend to be associated with greater satisfaction (75.67 and 77.71, *p* = 0.065).

**Table 7:** Mean scores for the BRECON-31 categories across the three study groups, obtained (one-way ANOVA test)

Category	Total	Group A	Group B	Group C	*p* value
Self-image (SD)	67.07 (± 17.31)	69.74 (± 15.68)	69.98 (± 16.73)	56.73 (± 18.8)	0.05
Arm concerns (SD)	75.8 (± 19.12)	80.95 (± 17.05)	77.16 (± 18.19)	66.25 (± 21.1)	0.065
Intimacy (SD)	75.76 (± 14.76)	74.73 (± 18.14)	80.05 (± 9.59)	68.18 (± 15.53)	0.079
Satisfaction (SD)	73.19 (± 21.3)	75.69 (± 17.13)	77.71 (± 20.79)	60.76 (± 24.58)	0.065
Recovery (SD)	54.47 (± 18.59)	61.56 (± 21.29)	50.21 (± 16.81)	52.36 (± 15.84)	0.107
Self-consciousness (SD)	66.8 (± 21.44)	76.19 (± 16.51)	68.66 (± 20.45)	50.55 (± 21.06)	< 0.001
Expectations (SD)	74.16 (± 14.34)	73.26 (± 14.66)	78.17 (± 12.38)	68.75 (± 15.88)	0.145
Breast appearance (SD)	67.48 (± 18.36)	67.06 (± 18.72)	72.33 (± 16.79)	59.99 (± 18.95)	0.119
Summary score (SD)	70.31 (± 14.49)	72.57 (± 12.47)	74.19 (± 12.87)	57.84 (± 15.97)	0.019

Group C had lower average scores compared to the other groups in all analyzed categories except for the Recovery category; however, these results reached statistical significance only for the Self-awareness category (*p* < 0.001) and the Total Score (*p* = 0.019). In addition, they showed a trend approaching significance in the categories Self-image *(p* = 0.05), Arm Concerns (*p* = 0.065), Intimacy (*p* = 0.079), and Satisfaction (*p* = 0.065) (Table 7).

## Discussion

A retrospective analysis was conducted to evaluate and compare three distinct approaches for direct-to-implant breast reconstruction. In our clinical practice, we have observed a shift in strategy that is consistent with the findings in the literature, where there has been a preference for the pre-pectoral technique over the submuscular approach [8, 20].

The findings of the second half of our study (since January 2022) indicate that pre-pectoral reconstructions were conducted in 23 out of the 27 cases (85.1%), while submuscular reconstructions without and with ADM accounted for 7 out of the 41 cases (17%) and 1 out of the 23 cases (4.3%), respectively. This change is justified by the improved learning curve in the use of ADM in our clinic and, consequently, by the promising results achieved with the pre-pectoral technique.

In their 2020 study, Caputo et al. examined the esthetic and functional outcomes associated with immediate submuscular and pre-pectoral breast reconstruction techniques in a cohort of 94 patients (121 breasts) [21]. The findings indicated that pre-pectoral direct-to-implant (DTI) reconstruction resulted in less impairment of upper-limb functionality and a decreased necessity for rehabilitation (74% vs. 91%). In addition, the perceived quality of life was marginally superior in the pre-pectoral reconstruction group, particularly in terms of sexual well-being and satisfaction, with a noted reduction in postoperative pain.

Cogliandro et al. conducted an analysis of the quality of life among 52 patients who underwent mastectomy followed by immediate implant-based reconstruction [22]. The study found that patients who received pre-pectoral reconstruction reported higher BREAST-Q scores compared to those who underwent submuscular reconstruction, particularly in the domains of psychological, sexual, and physical well-being, as well as satisfaction with the information provided. Furthermore, the pre-pectoral group exhibited significantly lower rates of postoperative complications and reoperation.

A multicenter retrospective study conducted by Ribuffo et al. examined postoperative complications and esthetic outcomes in two groups: 509 patients who underwent dual-plane reconstruction (characterized by a partially submuscular implant with acellular dermal matrix [ADM] coverage of the lower pole) and 207 patients who underwent pre-pectoral reconstruction utilizing ADM [23]. The findings indicated that the pre-pectoral group experienced a reduced incidence of complications, such as seromas and hematomas. Aesthetic outcomes, assessed by two independent plastic surgeons using a Visual Analog Scale (VAS) on standardized photographs, were more favorable for pre-pectoral reconstruction. The authors concluded that retro-pectoral reconstruction with ADM amalgamates the disadvantages inherent in both pre-pectoral and total submuscular techniques.

While additional studies conducted in 2024 have demonstrated the superiority of the pre-pectoral technique over submuscular techniques [24, 25], the comparative efficacy of these methods remains a subject of ongoing debate. In a 2021 study, Le et al. examined BREAST-Q scores to compare pre-pectoral and submuscular DTI reconstructions, finding that patient satisfaction levels were similar between the two groups; however, the subpectoral reconstruction group reported higher scores in the domain of sexual well-being [26]. Bekisz et al. conducted a study in 2021 involving 826 cases of prosthetic or expander-based reconstruction, assessing reconstructive outcomes and complications across three patient groups: total submuscular reconstruction (*n* = 392), dual-plane reconstruction (*n* = 358), and pre-pectoral reconstruction (*n* = 76) [27]. The overall complication rates were comparable among the groups. Notably, the dual-plane reconstruction group exhibited a higher incidence of infections and implant removal compared to the total submuscular reconstruction group, whereas the pre-pectoral reconstruction group experienced a higher rate of isolated wound dehiscence, seroma, and implant removal.

In our study, the incidence of minor complications was higher in the group undergoing submuscular reconstructions without ADM (26.8%) compared to the pre-pectoral (7.4%) and dual-plane (13%) groups (*p* = 0.086). This discrepancy may be attributed to the reduced tension on wound margins due to the presence of ADM in Groups A and B. However, these data may also be influenced by biases in reporting minor complications, which are often resolved through conservative outpatient treatments and may not be consistently categorized by the medical team conducting follow-ups.

The evaluation of major complication rates (*p* = 0.076) and reconstruction failure (*p* = 0.048) among the three groups demonstrated the superiority of the pre-pectoral technique (11.1% and 3.7%) and the submuscular technique without ADM (9.8% and 4.8%) compared to the submuscular technique with ADM (30.4% and 21.7%, respectively). The elevated rates of major complications, reconstruction failure, and capsular contracture associated with the submuscular technique using ADM may be explained by the increased risk of ADM-related complications, such as seroma and infection [28, 29], as well as potential issues related to pectoral muscle dissection [13].

The incidence of severe capsular contracture (Baker grades III–IV) was also significantly higher in Group C, which underwent submuscular reconstruction with ADM (21.7%), compared to the other two groups. This finding may be directly linked to the higher incidence of complications such as seroma and hematoma in Group C, which subsequently increases the risk of capsular contracture [30].

Although statistical analysis did not reveal significant differences in the distribution of anthropometric variables and pre- and perioperative risk factors among the three groups, it is noteworthy that Group C of dual-plane reconstructions had a higher rate of axillary lymph node dissections than the other two groups (33% versus 22.2% in Group A and 14.4% in Group B, *p* = 0.341). This suggests a possible influence of axillary dissection on poorer postoperative outcomes and lower BRECON-31 scores in Group C [31–33].

Group C also exhibited a higher rate of skin-sparing mastectomy (SSM) compared to the other two groups (48.1% in Group C versus 9.7% in Group A and 19.1% in Group B, *p* = 0.001). The clinical outcomes may have been affected by the large-volume resections inherent in these procedures, which may have contributed to the overexpression of cytokines and the inflammatory response. In contrast, large-volume mastectomy can indirectly favor excessive but involuntary thinning of the cutaneous flaps, promoting dense capsular formation and secondary breast distortion. Unfortunately, the literature does not currently support these hypotheses, ascribing a higher risk of complications to NSM than SSM; however, data originating from solid stratification between patient demographics and type of mastectomy are still to be shown.

Patient satisfaction and quality of life were analyzed using the BRECON-31 questionnaire. In the categories “Arm Concerns” and “Recovery,” the group of patients undergoing pre-pectoral reconstruction showed higher scores compared to the other two groups, with values of *p* approaching significance only for the former (*p* = 0.065). This confirms that avoiding dissection of the pectoral muscle leads to better postoperative upper-limb functionality and less postoperative pain with quicker recovery [34].

The categories “Self-Image” (*p* = 0.05), “Satisfaction” (*p* = 0.065), “Breast Appearance” (*p* = 0.119), and “Overall Score” (*p* = 0.019) showed similar results for the pre-pectoral and submuscular without ADM reconstruction groups, while the dual-plane group had lower scores on average. The “Self-Awareness” category showed superiority of the pre-pectoral reconstruction group over the other two groups (*p* < 0.001). Overall, in all categories except “Recovery,” the group of patients undergoing submuscular reconstruction with ADM responded with lower scores compared to the other two groups.

In 2022, Bassetto et al. [35] published a study on the treatment of capsular contracture in breast reconstructions with implants fully covered by ADM. The study found a lower recurrence of contracture in patients with ADM-covered implants compared to those without ADM coverage. However, the ADM group had higher rates of implant removal, minor complications, red breast syndrome, and skin ulceration. Despite these complications, the ADM group reported higher scores in “Satisfaction” and “Physical Well-being.” The authors concluded that while ADM coverage may reduce capsular contracture recurrence, its use should be reserved for selected patients to minimize complications and enhance patient outcomes.

The present study is subject to several limitations that merit consideration. First, the exclusive use of a single type of acellular dermal matrix (ADM) precludes a comprehensive comparison across different biological prostheses. Second, the lack of pre- and post-mastectomy measurements of skin flap thickness hinders the evaluation of the surgical procedure’s aggressiveness. Third, the limited sample size restricts the ability to achieve statistically significant *p*-values for each factor. In addition, the unequal distribution of patients across the three groups diminishes the statistical robustness of the findings. Lastly, partial adherence to the Brecon-31 questionnaire by patients further constrains the study’s conclusions.

## Conclusions

Prepectoral implant-based reconstruction, along with traditional submuscular techniques, has proven to be effective in the context of direct-to-implant (DTI) breast reconstruction; however, the pre-pectoral technique with ADM does not seem to have superiority over the conventional muscle-pocket technique in terms of reducing complication rate and overall patient satisfaction.

Some concerns about questionable vascularity of mastectomy skin flaps arose from this study in cases of skin-sparing mastectomy (SSM) in large breasts. In such cases, surgeons should carefully reflect on the potential risk of wound dehiscence and implant infection, considering the shift in reconstructive planning towards a two-stage reconstructive approach using tissue expanders or autologous techniques.

The application of the dual-plane technique with implants wrapped by ADM has not revealed any benefits in immediate breast reconstruction, even though recent findings suggest that it may have utility in addressing complex secondary cases, especially in the management of severe capsular contracture over the long term.

## Data Availability

All datasets generated during and/or analyzed during the current study are included in this published article.
